# Remediation Effects on N170 and P300 in Children with Developmental Dyslexia

**DOI:** 10.3233/ben-2009-0257

**Published:** 2010-06-29

**Authors:** Mélanie Jucla, Rodolphe Nenert, Yves Chaix, Jean-François Demonet

**Affiliations:** ^1^InsermImagerie cérébrale et handicaps neurologiques UMR 825ToulouseFrance; ^2^E.A Octogone. Laboratoire Jacques-LordatE.A 4156Université Toulouse II Le MirailToulouseFrance; ^3^Universite de Toulouse; UPS; Imagerie cérébrale et handicaps neurologiques UMR 825; CHU PurpanToulouse Cedex 9France; ^4^Centre Hospitalier Universitaire de ToulousePole NeurosciencesCHU PurpanToulouse Cedex 9France

**Keywords:** Developmental dyslexia, remediation, children, phonological, visual attention, ERPs

## Abstract

This study aimed at investigating the ERP correlates (N170 and P300 components) of a multimodal training program focused in dyslexia. ERPs were obtained from 32 electrodes in 24 French children with developmental dyslexia (mean age 10 years 7 months) during a visual lexical decision task. All the children received two intensive two-month evidence-based training programs: one based on phonemic awareness and the other on visual and orthographic processing in a cross-over design. Ten control children matched on chronological age were also tested. We showed dissociation between N170, P300 and behavioral improvement. In the dyslexic group, P300 amplitude decreased for non-words and words as the latter yielded performance improvement. In the control group, the same effect was observed for pseudo-words. At the same time, the opposite pattern occurred for the N170 latency, which was shortened for pseudo-words and pseudo-homophones in the dyslexic group and for words in the typically achieving children. We argue that training might modulate cortical activity in dyslexic children in a visual word recognition task. Considering the well-known implication of P300 in attentional processes, our results reflect the strong link between reading skill improvement after remediation and visual attentional process maturation.

## 1. Introduction

Dyslexia, or reading difficulty, is one of the most common problems that severely affect academic performance. It is revealed as a difficulty in learning to read despite conventional teaching, without being the direct result of any intellectual disadvantage or unfavorable environmental influence [2]. It is still not precisely known what factors prevent normal acquisition of reading abilities, as reading is a complex phenomenon. In the last 20 years, the most convergent evidence has arisen from the phonological deficit hypothesis, according to which the difficulties suffered by dyslexic children in identifying words in a text are due to deficiencies in their ability to segment the flow of speech into its sound components, also known as phonological awareness [33,47–50]. However, alternative explanations have been suggested, implicating visual attention [15]. Recently, Valdois and collaborators [4] found a link between visuo-attentional span and reading process. The authors suggest that phonological and visuo-attentional skills are implied in reading efficiency, considering that their alteration results in poor performance in tasks that permit developmental dyslexia to be characterized.

Several remediation programs have been suggested for dyslexia, each of them focusing on one 'core' deficit. Several studies have shown significant improvement in reading skills using remediation programs based on phonological processing only [18] (see Beaton for a review [2]) or instruction combining phonic coding and literacy skills [25,54]. Magnan and collaborators used audiovisual software to train dyslexic children in grapheme-phoneme correspondence rules [26,27]. They found significant improvement in word recognition after training. However, Alexander and Slinger-

[1]These authors contributed equally to this work.

ISSN 0953-4180/09,10/$27.50 © 2009/2010 – IOS Press and the authors. All rights reserved

Constant underlined the fact that, despite improvements observed after training, a substantial number of dyslexic children resist treatment, whatever the remediation modality [1].

The event-related potential (ERP) technique has allowed researchers to decompose the cognitive process of word reading into temporal phases. With regard to our interest in developmental impairment, two crucial components have been identified:

The first peak around 170 ms after the stimulus onset (N170 hereafter) is thought to reflect the first cognitive orthographic process with a source in occipito-temporal regions [22,37]. Some authors infer that this component reflects the first letter-specific treatment compared to that of any other symbol [41]. In addition, its amplitude is larger after word or even pseudo-word (i.e. pronounceable meaningless letter sequence) than nonword (i.e. illegal letter sequence as for instance string of consonants) presentation, suggesting a pre-lexical component [3,8,28,30,32,36]. However, this component has been found to be modulated by word lexical frequency [45,46]. These latter works suggest that N170 may also reflect lexical access in single word reading. This component has been found to differ in dyslexic subjects compared to controls in several studies, generally revealing a higher latency and lower amplitude in dyslexic subjects [19,41,42,51,52]. N170 amplitude increase during reading learning seems to characterize reading level. In a longitudinal study, Maurer et al. showed that N170 amplitude increased with learning and that this effect was reduced in children with reading impairment [29].

The second component is a positive wave that occurs around 300 ms after stimulus presentation (P300 hereafter) and is known to reflect the amount of attentional resources involved in a task [21,57]. However, considering written word processing, it has been found that word recognition elicits a higher P300 [40] and a lack of P300 when subjects are stimulated with pseudo-homophones or words with internal case changes [44]. Moreover, Duncan et al. observed P300 anomalies in adults with dyslexia who had also suffered from attentional disorder during childhood [12]. Thus, considering that attentional disorders are frequently observed in dyslexic subjects, it is difficult to resolve whether P300 anomalies are only due to dyslexia or associated with attention disorders [53].

Few studies address the question of the consequence of remediation on the temporal course of written word processing in dyslexia. Recently, Santos et al. studied the effects of a training program based on both phonemic awareness and grapheme-phoneme correspondence exercises [9,17,18] on auditory language semantic integration [43]. Before training, ERPs (between 200 and 700 ms) were modulated by incongruity in the typically achieving children only. After training the same effect was observed in the dyslexic group.

The aim of the present paper is to study the ERP correlates of a multimodal evidence-based remediation program that focused on two major deficits in dyslexia, phonological and visual attentional. With this is mind, we analyzed two crucial components, the N170 and P300 in a lexical decision task using a typical Oddball paradigm in dyslexic children that followed our program compared to typically achieving children as a baseline. We hypothesized that control subjects should show larger N170 and P300 with shorter latencies than dyslexic children and that the latter should present a more typical pattern of activity after remediation.

## 2. Methods

### 2.1. Participants

24 French-native-speaker dyslexic children from 9 to 11 years old (9 girls and 15 boys) were recruited. All subjects had an IQ $>$ 80 as tested with WISC-III [56] and a reading delay of at least 18 months as tested by a standardized French reading test (L'Alouette [24]). All subjects satisfied developmental dyslexia diagnosis criteria according to ICD-10 classification (WHO, 1993). They had no specific attentional disorder as tested by the D2 test [5], no oral language delay as tested by L2MA [6] and TCG [10] and no comprehension disorder as tested by ECOSSE [23].

In addition, 11 French-native-speaker control children from 9 to 11 years old (4 girls and 7 boys) were also recruited. They had no IQ deficit or reading level delay, as tested by WISC-III [56] and the "Alouette" tests [24] respectively. Nor chronological age neither IQ differed between the control group and the dyslexic group (resp. $p = 0.15$ and $p = 0.14$).

### 2.2. Remediation program

Training sessions were implemented six days a week and lasted from ten to twenty minutes each day. Children were assisted by their parents at home or by their speech therapist, who switched from the usual therapy to these programs. Before each training period, specific material (stimuli and exercises, audio CD and notebooks) was delivered to the parents and detailed explanations and examples were provided (written directions were also given). Adults were asked to provide feedback to the child, especially in case of erroneous responses. They were taught how to use the material and what instructions and feedback to give the child during a one-hour training session. After each training period, exercise notebooks were collected and reviewed, and a debriefing with the parents and the speech therapist was held in order to check the reliability of the interventions.

Data from one child were discarded because of lack of precision and compliance in the training program (this child was not included in the sample described above).

*2.2.1. The phonological training*

This training was derived from that used in Habib et al. [18]. Stimuli were presented through headphones from an audio CD. The adult had to give the instructions and take note of the child's answers in an exercise book. There were 6 exercises per day. The typical pattern of exercises was different between weeks 1, 3, 5 and weeks 2, 4, 6.

Exercises proposed during weeks 1, 3 and 5 were as follows:

The first three exercises contained triplets of words the child had to compare phonologically to disclose similarity between 2 of the 3 stimuli, either in the rhyme, the onset, or the middle part of words (for example, "which words rhyme among the triplet "boat", "coat", "bowl"?"). The other exercises consisted of syllable counting, phoneme detection ("How many times can you hear the sound /s/ in 'sausage'?") and finally, word repetition.

Exercises proposed during weeks 2, 4 and 6 were as follows:

The items were pseudo-words during weeks 2 and 6, and words during week 4. In the first two exercises, the children were asked to find the odd word among three pseudo-words or words. The oddity was based on the rhyme (exercise 1) or the onset (exercise 2). In the third exercise, the children had to find a target phoneme in one of the three pseudo-words or words. Then a phoneme counting task was proposed followed by spoonerisms (based on words). Finally the child had to repeat pseudo-words or words.

*2.2.2. The visual training*

The visual training was split into two different parts.

The first 3 weeks consisted of non-verbal visual attentional training while the second 3 weeks consisted in a shift of visual attention and perception exercises from the non-verbal to the verbal domain. The first, non-verbal phase was meant to provide initial training so that children could become familiar with heavy-duty exercises on visual/graphic stimuli. The second phase also involved visual stimuli but they consisted of orthographic material.

The first-phase, non-verbal exercises were derived from various tests and rehabilitation sets used in speech therapy for dyslexia remediation. The exercises mainly focused on:

– Space organization: path finding through a maze-like design, drawing a path by following arrows or between an arrangement of points identical to that represented on a model,
– Visual attention: identifying superposed geometrical forms, matching a drawing to sample, coding, matching nonsense figures by sticking them on the model, superposing geometrical figures with their matching model drawn on a transparency sheet.
– Logic: matrix completion tasks.

Responses were checked and corrected by the parents or the speech therapist.

The second 3-week period was a specific visual orthographic training that had been built up for a previous study in our laboratory with the cooperation of speech therapists (see typical examples of exercises in Appendix C). The goal of this part was to enhance access to orthographic knowledge and lead subjects to work out and visualize word orthography mentally. The exercises mainly focused on:

– Shape of letters and words: for each letter in an orally presented word, saying if it went up (e.g. "d"), down (e.g. "p") or in the middle (e.g. "e"); identifying if a "shadow envelope" could hide a target word.
– Searching for a word or a letter: identifying a word embedded in a string of letters; chunking a text presented without spaces; linking spread letters according to a model word; letter cancellation task.
– Orthographic knowledge: spelling out or counting the letters in an orally presented word and writing it down; naming the letters situated just before and after a target letter in an orally presented word.
– Word comparisons: similarity judgment task in word pairs; choosing which of four choices was identical to the model; finding the missing letter in a word; identifying the longest word.
– Reading a word presented in an unusual manner: vertically; around a circle.
– Converting a non-word presented in upper case letters into lower case or the contrary.

### 2.3. Procedures

In order to avoid any order effect in remediation, 12 dyslexic children first followed the phonological remediation then the visual remediation. The other 12 dyslexic children started with the visual remediation then followed the phonological remediation. Children were included in one of the two groups by alternation. All children underwent EEG recording twice: before the training (Session 1) and after training (Session 2, 19 weeks after session 1).

During each EEG session, ERPs were obtained for each child, who had to complete a visual lexical decision task. This task involved 128 items that were distributed over 4 conditions in an Oddball paradigm: 32 words, 32 pseudo-words (pronounceable meaningless sequences), 32 pseudo-homophones (same phonology as, different spelling than a real word) and 32 non-words (unpronounceable letter sequences). This particular stimuli distribution was made to elicit maximum subject attention over word stimuli. Three lists of 128 items were created in order to have different stimuli in each EEG session; these lists were balanced for lexical frequency, number of letters, and syllable structure. List order was counterbalanced between subjects.

Subjects were comfortably seated in front of a screen in a quiet room. During the task, a centrally located fixation cross stimulus first appeared for 500 ms, then one item appeared for 250 ms, followed by a fixation cross again. Inter-stimulus interval varied between 1700 and 2000 ms. The subjects were explicitly asked to indicate whether the sequence presented was a French word by pressing one of two designated keys using both hands. The use of the right versus the left index to designate real words was alternated between subjects.

### 2.4. ERP acquisition

The electroencephalogram (EEG) was recorded with a 0.1–100 Hz filter and a 500Hz sampling rate using Neuroscan 4.2 software and a 32-electrode Neuroscan device (Ag-AgCl electrodes). Impedances for all electrodes were kept below 5 kohms. The vertex electrode was used as the recording reference. Eye-movements were monitored with two electrodes: one placed above the left eye and one placed on the right temple.

The epochs contaminated by eye-movements or artifacts of non-biological origin producing voltages larger than $+/-$ 125 $\mu$V peak-to-peak were omitted from averaging.

The ERPs were digitally filtered with a low-pass filter of 30 Hz (12dB/Octave, zerophase-type filter) and a high-pass filter of 0.5 Hz (12dB/Octave, zerophase-type filter).

The analysis epoch began 100 ms before and terminated 1200 ms after stimulus onset.

A baseline correction was applied from $-100$ ms to 0 ms. Average reference was applied to all ERPs.

### 2.5. Analyses

#### 2.5.1. Behavioral statistics

Data from one dyslexic child was discarded due to a problem in response recording. An ANOVA with repeated measures was carried out on number of correct answers (accuracy rate) with Session (session 1 and 2) X Condition (words, pseudo-words, pseudo-homophones and non-words) as intra-individual factors and group (experimental group and control group) as inter-individual factor.

#### 2.5.2. ERP statistics

In a first step, we conducted an automatic peak research on defined time windows (using the Global Field Power (GFP) of 32 electrodes on average waves in the control group [35]): [150–250] ms for N170 and [250–400] ms for P300.

Data from two dyslexic children were discarded due to a problem in signal recording. Peak mean amplitudes and latencies were analyzed using ANOVA with repeated measures on the electrodes that showed the highest sensitivity: P7, P8, PO9, PO10, O1 and O2. Session (before/after training), condition (non-word, pseudo-word, homophone and word), hemisphere (right/left) and electrode were considered as intra-individual factors. Group (dyslexic versus control) was considered as an inter-individual factor. To clarify the results described below, we have chosen not to report main effect or interactions involving the 'electrode' factor.

## 3. Results

### 3.1. Behavioral results

Raw scores (and SD) are presented in Table 1. Analysis on the accuracy rate revealed a significant group effect (F(1,32) $=$ 5.65, $p = 0.023$), dyslexic children being less efficient that control children. Both groups showed a significant improvement between two sessions (F(1,32) $=$ 4.71, $p = 0.037$). Performance was

Table 1
Results obtained (% of accuracy) in different conditions of the visual lexical decision task in dyslexic and control group. S1 stands for session 1 (resp. S2 for session 2). Standard deviation is in brackets

| Group | Pseudo-homophones | | Non-words | | Words | | Pseudo-words | |
|---|---|---|---|---|---|---|---|---|
| | S1 | S2 | S1 | S2 | S1 | S2 | S1 | S2 |
| Dyslexic | 44.7 (22.2) | 48.0 (21.1) | 68.0 (26.5) | 69.5 (26.3) | 55.3 (24.6) | 63.6 (20.2) | 51.4 (23.7) | 55.7 (19.1) |
| Control | 56.5 (24.0) | 60.1 (24.5) | 82.1 (21.0) | 84.6 (20.0) | 75.3 (17.9) | 70.3 (21.1) | 65.2 (21.8) | 80.8 (18.2) |

affected by condition (F(3,96) = 15.72, $p < 0.001$). A post-hoc Tukey test showed that the non-word condition was the easiest (significant difference between non-words and words – $p < 0.05$ – and both types of pseudo-words – $p < 0.001$) and pseudo-homophones were the hardest (difference between pseudo-homophones and words, non-words – $p < 0.001$ – and pseudowords – $p < 0.05$). Session x Group or Condition x Group interactions were not significant whereas a Session x Condition x Group interaction was significant (F(3,96) = 2.76, $p = 0.046$). A post-Hoc Tukey test showed that, in the control group, in session 1, pseudo-homophones were significantly less well identified than words ($p < 0.05$). This difference disappeared in session 2. In the dyslexic group, words were significantly less well identified than non-words in session 1 ($p < 0.05$). This difference disappeared in session 2 due to an improvement in word identification.

## 4. ERP results

Curves are depicted in Fig. 1.

N170: Peak latency analysis showed that only the Session x Group x Condition interaction was significant (F(3,93) = 3.97, $p = 0.01$). According to the post hoc analysis (Fisher LSD test), latency was found to be smaller in session 2 compared to session 1 for non-words in the control group ($p < 0.005$) and for pseudo-words in the dyslexic group ($p = 0.06$).

Peak mean amplitude analysis also showed that only the Session x Group x Condition interaction was significant (F (3,93) = 2.87, $p < 0.05$), the amplitude being smaller in dyslexic children for pseudo-words and pseudo-homophones after training (Tukey test; resp. $p < 0.001$ and $p < 0.005$).

P300: Peak latency analysis showed that only the Session x Group interaction was significant (F(1,31) = 4.2, $p < 0.05$), control children having smaller latency in session 2 (Fisher LSD test; $p = 0.06$).

Peak mean amplitude analysis showed a Condition main effect as a Condition x Hemisphere significant interaction (respectively F (3,93) = 4.6, $p < 0.005$ and F (3,93) = 5.23, $p < 0.005$). The mean amplitude was higher for words compared to both types of pseudo-words (Tukey test; $p < 0.001$) and non-words ($p < 0.05$) in the left hemisphere only. Session X Condition X Group interaction was – gnificant (F (3,93) = 4.08, $p < 0.01$), showing an amplitude decrease in the control group for both types of pseudo-words ($p < 0.005$) and in the dyslexic group for words and non-words ($p < 0.001$) in session 2 compared to session 1.

## 5. Discussion

In this study we have investigated the neural correlates of reading improvement after intervention in developmental dyslexia. We analyzed dyslexic children's ERPs elicited by a lexical decision task before and after a four-month phonological and visual orthographic training program. We compared their data to those of typically achieving children as a baseline before and after the period of four months. We were interested in the main effects of group, task condition, session and interactions between these parameters.

If we first look at the main group effects, not surprisingly, dyslexic children were found to have poorer behavioral performance on the visual lexical decision task. This supports the orthographic encoding deficit in dyslexia [11].

We also highlighted main condition effects in behavioral and electrophysiological data in both groups. First, considering accuracy scores, the non-word condition was easier to address than the pseudo words, pseudo-homophones and even words. According to the DRC reading model, processing an unpronounceable letter sequence and deciding it is not a word is faster because one does not have to processes more than orthotactic information [7]. For pseudo-words, the subject has to activate a phonological content and then may check whether it activates semantic information. Furthermore, the latter could be responsible for the specific difficulty in addressing the pseudo-homophones. Processing a homophone yields a mismatch between the current orthographic visual input and the orthographic information that is sent back after the corresponding semantic contents have been activated. While this ef-

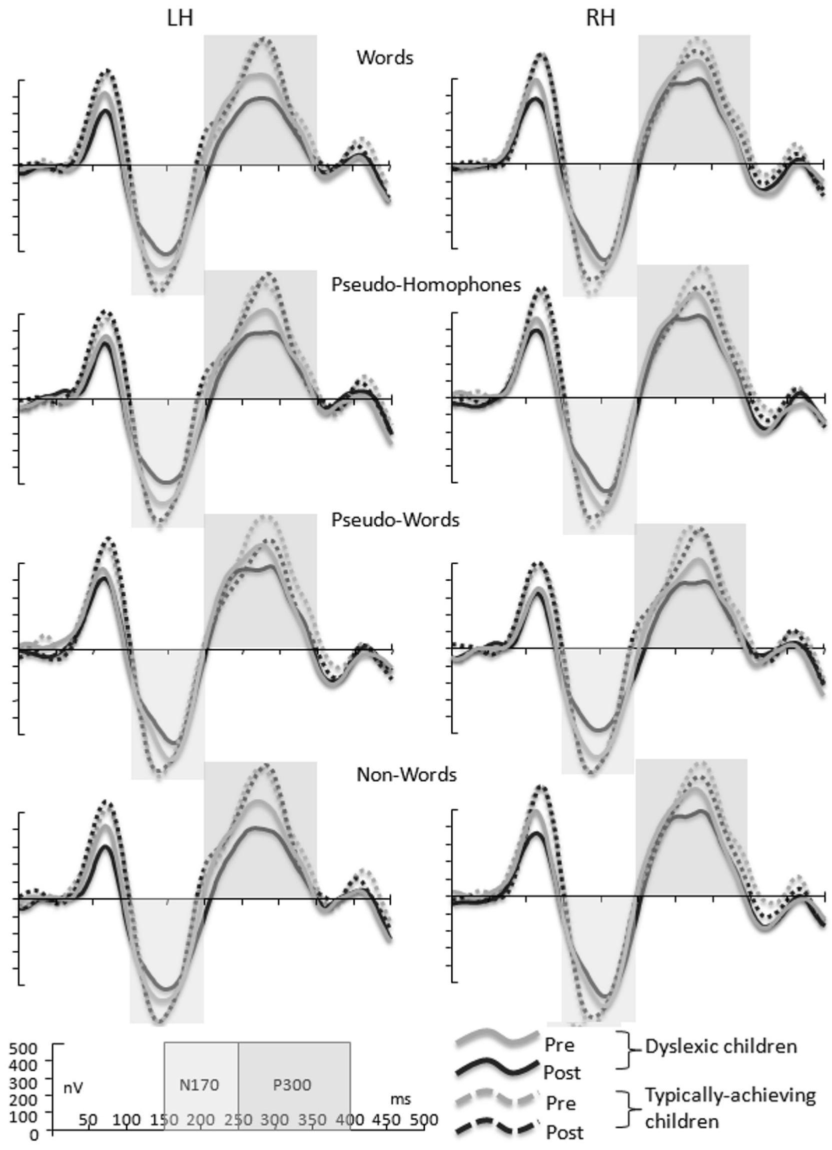

Fig. 1. ERP curves obtained from the linear derivation of P7, PO9 and O1 for the left hemisphere (LH) and P8, PO10 and O2 for the right hemisphere (RH) in the different conditions of the visual lexical decision task at session 1 and 2 (pre/post) in both groups of children.

fect is crucial during reading acquisition, it is known that the orthographic lexicon is not stable at the maturational stage [16] corresponding to the children we studied (mean chronological age 10 years).

Secondly, looking at the ERPs results, we observed that words elicited a higher P300 amplitude than other stimuli (non-words and pseudo-words) in both groups. This finding may be interpreted as a typical attentional effect when target occurrence is less frequent than distracters [34,38]. In our paradigm, real words only represented 25% of items. However, this difference only occurred in the left hemisphere, showing a possible supplementary attentional load due to lexical treatment in this hemisphere [14].

We could not demonstrate a main session effect but complex session x condition x group interactions were found in behavioral and electrophysiological results (see Table 2). Both groups showed improvement in the task and a decrease of N170 latency and P300 mean amplitude. As the control group did not receive any specific training, this could easily be attributed to procedural learning of the task. Nevertheless, the specificity of these modulations of activity according to the group

Table 2
Effects distribution observed between first and last session

| Observed effect | Dyslexic group | Control group |
|---|---|---|
| Recognition improvement | Words | Pseudo-homophones |
| N170 latency decrease | Pseudo-words | Non-words |
| N170 amplitude decrease | Pseudo-words and pseudo-homophones | / |
| P300 amplitude decrease | Words and Non-words | Pseudo-words and pseudo-homophones |

and condition suggests a more complex interpretation of these findings.

A relationship between behavioral and electrophysiological changes seems to emerge, showing dissociation between N170, P300 and behavioral changes (see Table 2). In the dyslexic group, P300 amplitude decreased for non-words and words whereas the latter yielded performance improvement. In the control group, the same effect was observed for pseudo-words. At the same time, the opposite pattern occurred for the N170 latency, which was shortened for pseudo-words and pseudo-homophones in the dyslexic group (the latter result being associated with a decrease of the peak mean amplitude) and for words in the typically achieving children without any correlates on behavioral results. These results raise two questions. First, why would electrophysiological change be related with behavioral improvement only for the P300 component? Second, why did we observe an opposite condition effect depending to the group?

In this study, we used a lexical decision task in an oddball paradigm in order to investigate both orthographic and attentional processing. It appeared that the task required heavy attentional load due to short presentation time. As shown in Table 1, an important variance in the performance of the two groups was observed. Taking this into account and the variance in electrophysiological data, we might be facing a basic problem of statistical power that could not allow showing more effects. Therefore, due to the attentional demand required by the task, P300 effects might be more robust than N170 ones. It would be of interest to further investigate our hypothesis on the N170 component on a classic lexical decision task with a greater group of subjects. Moreover, we argue that a possible behavioral improvement in this specific task would more rely on attention than orthographic processing skills. In their remediation study, Santos et al. [43] put forward ERP change in the P300 component after an audiovisual training program in dyslexia in a pitch incongruity perception task. They argue that the training provides better skills in detection tasks. Furthermore, other learning studies have shown a great sensitivity of the P300 component to cognitive changes occurring after training [20,31,39].

Moving to our second question, one may wonder whether the changes observed in the two groups, with opposite effects of conditions, rely on the same processes in both groups. We argue that the mechanisms involved are different in the dyslexic compared to typically achieving children. In the control group, it may be suggested that procedural learning accounted for an improvement of performance on pseudo-words; this processing facilitation might account for P300 amplitude decrease as a result of decreased attentional load. This effect would only appear for the pseudo-words as performance on identification of both non-words and words in this group was already high at session 1 (resp. 82% and 75%) while subjects were less accurate for pseudo-words and pseudo-homophones (resp. 65% and 56%). Concerning dyslexic children, if their improvement had been only related to procedural learning, we would have expected performance increase to be independent of condition; and these subjects were deficient in all conditions compared to their peers. Instead, the training received might be at least partly responsible for the observed difference between conditions. The training was built, first, to improve visual attention and phonemic awareness and, second, to enhance the lexical route for reading, which permits good literacy skills in proficient pupils [13]. From orthographic representation to phonological coding, the children in the present study were taught how to implicitly use this route. According to the visual attention span deficit theory [55], word recognition requires a higher attentional level in dyslexic children, especially when they are confronted with long orthographic sequences [4]. So the decrease of P300 amplitude in the word condition may result from a reduced allocation of attention resources to these stimuli because of a better word identification (a specific improvement in reading process).

## 6. Conclusion

In this study, we have shown that cortical activity in dyslexic children in a visual word recognition task might be modulated by training.

However, only P300 modifications were related to behavioral improvement, both in dyslexic and in control subjects. Considering the well-known implication of P300 in attentional processes, our results reflect the strong link between reading skill improvement after remediation and visuo-attentional process maturation.

## Acknowledgement

We would like to thank Margot Taylor, Magali Batty, Marianne Latinus, Chloé de Boysson, Maria Trabanino, Stéphanie Iannuzzi, Evelyne Calvet, and Isabelle Barry for their help in the preparation and execution of this experiment. In its first phase, this research was supported by the INSERM program "PROGRES" and by a grant from the Clinical Research Hospital Program from the French Ministry of Health (PHRC 2002, University Hospital of Toulouse N° 0203208) and then by the "ARS 2000" program of the French Foundation for Medical research ("Fondation Recherche Médicale").
